# Hepatic abscess in a pre-existed simple hepatic cyst as a late complication of sigmoid colon ruptured diverticula: a case report

**DOI:** 10.1186/1752-1947-2-83

**Published:** 2008-03-14

**Authors:** Maria Chatzipetrou, Efthimios Avgerinos, Efstratios Christianakis, Galinos Barmparas, Nikolaos Pashalidis, Athanasios Stathoulopoulos, Panayiotis Tsatsoulis, Dimitrios Smailis, Dimitrios Filippou

**Affiliations:** 1 2ndDepartment of Surgery, "Asklepeion Voula's Hospital", Athens, Greece; 2 1stDepartment of General Surgery, Piraeus General Hospital, Athens, Greece; 3 Department of Anatomy, Nursing Faculty, University of Athens, Athens, Greece

## Abstract

**Introduction:**

Hepatic abscesses have been reported as a rare complication of diverticulitis of the bowel. This complication is recognized more commonly at the time of the diagnosis of diverticulitis, or ruptured diverticula, but also can be diagnosed prior to surgery, or postoperatively.

**Case presentation:**

This report describes a man who developed an hepatic abscess within a simple hepatic cyst, two months after operation for ruptured diverticula of the sigmoid colon. The abscess was drained surgically and the patient made a complete recovery.

**Conclusion:**

The development of an hepatic abscess in a pre-existing hepatic cyst, secondary to diverticulitis, is a rare complication. A high degree of clinical suspicion is required for immediate diagnosis and treatment.

## Introduction

After the advent of antibiotics, few cases of hepatic abscesses secondary to diverticulitis have been described. In the past pylephlebitis, with or without hepatic abscess formation, was a complication of abdominal sepsis more commonly due to appendicitis. Abdominal computed tomography (CT) scans have contributed to the early diagnosis of this lethal complication that had been mostly a postmortem finding in the past. Herein, we report the case of a man who developed an hepatic abscess within a pre-existing simple hepatic cyst, following surgery for ruptured diverticula of the sigmoid colon.

## Case presentation

A 66-year-old male was referred to our clinic with an acute abdomen by the cardiology unit, where he had been admitted three days before to be treated for paroxysmal supraventricular tachycardia (SVT) and Right Bundle Branch Block.

The patient was pale with tachycardia and tachypnoea. His temperature was 38.5°C. On physical examination there was localized tenderness at the left iliac fossa. Digital rectal examination was normal.

**Figure 1:**
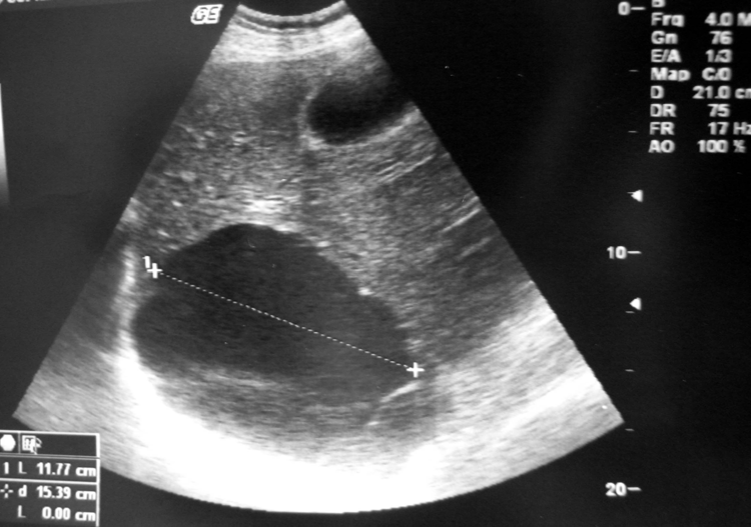
During first admission. Abdominal ultrasound revealing a sizeable right liver lobe cystic mass.

Blood tests on admission revealed: WBC: 18.200/mm^3 ^(Seg 90%, Lymp 7%, Eos 1%, M 2%), Ht: 35.2%, Hb: 11.5 g/dl and PLT: 537.000/mm^3^, AST/ALT: 35/29 U/l, LDH: 116, CPK 77. Ultrasound (U/S) and CT scan of the abdomen demonstrated multiple simple hepatic cysts on both lobes, the larger of those on the right lobe with a dimension of 12 cm. (Fig. 1)

After starting antibiotics (ticarcillin-clavulanic acid, amicacin and metronidazole), the patient was operated on two days later for peritonitis. At laparotomy, a small abdominal abscess was found at the area of the sigmoid colon caused by ruptured diverticula. The abscess was drained and Hartmann excision of the bowel was performed. The patient was admitted to the ICU for the first four postoperative days and was discharged fully recovered on the 21st day postoperatively from the clinic. Cultures from the abdominal abscess grew *Aerococcus viridans*, *Escherichia coli *and *Proteus mirabilis *and were negative for anaerobes. Antibiotics were administrated for 18 days after the operation. After discharge only dietary restrictions were recommended.

**Figure 2:**
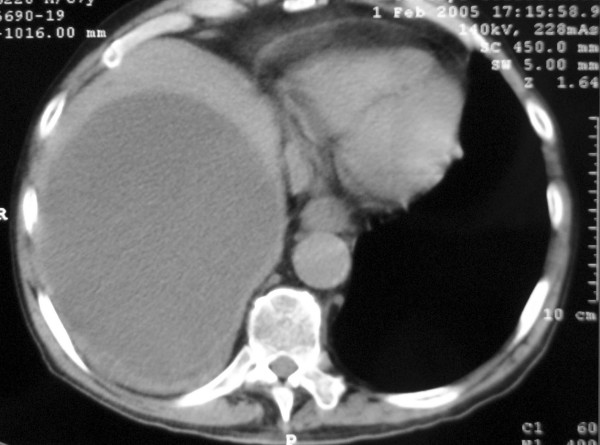
During second admission. Abdominal CT scan revealing the same clear right liver lobe cystic mass.

One month later the patient returned to the hospital with malaise, vague abdominal pain, anorexia, poor general condition and slightly elevated temperature (37.2–37.5°C). Physical examination of the abdomen was normal, and the laboratory results were: WBC: 10.100/mm^3 ^(Seg 78%, Lymp 16%, Eos 2%, M 4%), Ht: 29.1%, Hb: 9.3 g/dl and PLT: 629.000/mm^3^, AST/ALT: 126/37 U/l, amylase 184. Abdominal ultrasound and CT scan did not reveal any abnormal or unexpected finding, except of course the simple hepatic cysts which were of the same dimensions. (Fig. 2)

**Figure 3:**
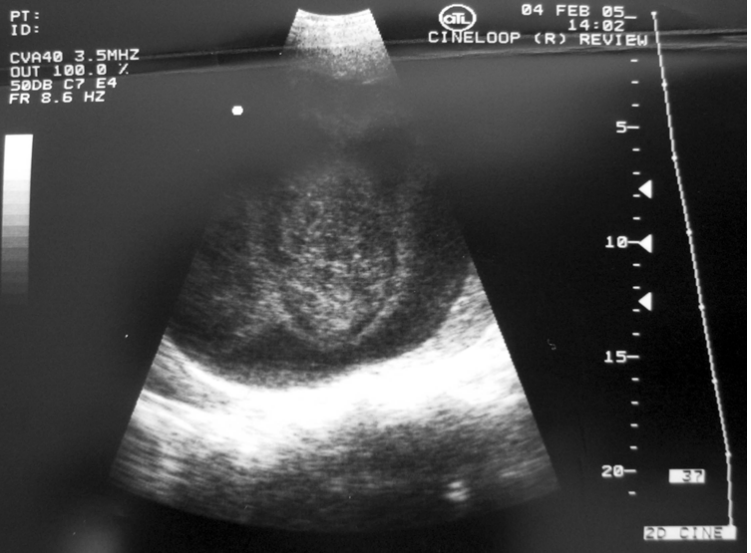
During second admission – 3 days post-admission. Ultrasound revealing the opaque fluid collection (abscess) inside the previously clear right liver lobe cystic mass.

Three days after his admission, because of a drop of the hematocrit to 20.9%, elevation of the patient's temperature (39°C), and worsening of his general condition, ultrasound examination was repeated and showed that the largest of the hepatic cysts contained opaque fluid (Fig. 3), in contrast to the ultrasound that was performed at the first admission, where the fluid of the cyst appeared to be clear. Abdominal CT scan, showed the formation of an hepatic abscess at the site of the pre-existing hepatic cyst.

Due to an inability to drain the abscess under CT guidance at our hospital, the liver abscess was drained surgically. The cultures of the pus of the hepatic abscess grew *Escherichia coli *and *Enterococcus spp*. The postoperative care of the patient was uncomplicated and he recovered slowly but fully. He was discharged one month postoperatively.

## Discussion

In the pre-antibiotics era, rare but lethal cases of pylephlebitis and hepatic abscesses secondary to appendicitis had been described [1,2] but after the advent of antibiotics this complication has been observed more commonly after diverticulitis [3]. Later in the clinical course, infection of the liver can cause the formation of hepatic abscesses. Only a few cases of hepatic abscesses due to diverticulitis have been reported, with [4,5] or without coexistent pylephlebitis [6].

Our report concerns a unique case of hepatic abscess formation due to diverticulitis, in an existing simple hepatic cyst without evidence of pylephlebitis. This is the first reported case of delayed hepatic cyst transition to abscess, following an event of diverticular rupture. The cyst was first detected as a random finding during an abdominal CT scan during the first hospital admission of the patient. Later, during his second admission, the cyst was revealed again by U/S and was then found to have been infected and transformed to a large abscess three days later. That happened probably by migration of microbes via the portal vein but much later than the initial infection which took place two months earlier.

Gram negative and anaerobic organisms are most commonly isolated in blood cultures and specimens of abscesses, with *E. coli *being the most prevalent microbe [7,8]. That was also the case in our patient.

Hepatic abscess may precede [9], follow [10] or appear at the same time as diverticulitis [7,8]. Abdominal CT scan and sonography are essential diagnostic methods and one must have always in mind this rare but serious complication when dealing with diverticulitis of the bowel. Vice versa, one must remember the possibility of diverticulitis in the presence of hepatic abscesses [11].

In our case, we were able to document that the abscess of the liver appeared two months after the rupture of sigmoid colon diverticula. In spite of the high mortality related to this complication, our patient made a full recovery following surgical drainage.

## Conclusion

Hepatic abscess in a pre-existing hepatic cyst, occuring secondary to diverticulitis, is a rare complication and a high degree of clinical suspicion is required for immediate diagnosis and treatment.

## Competing interests

The author(s) declare that they have no competing interests.

## Authors' contributions

CM, BG, PN, SA, TP, FD, AE, CE were the attending physicians as well as the coordinating doctors (microbiologist, radiologist) who were responsible for the diagnosis and treatment of the patient and who provided all the information. CM, SD, TP, BG, helped to draft the manuscript. CE, SA, PN, FD wrote the final manuscript. AE, SD, FD performed the final revisions of the paper. All authors read and approved the final manuscript.

## Consent

Written informed consent was obtained from the patient for publication of this case report and accompanying images. A copy of the written consent is available for review by the Editor-in-Chief of this journal.
